# 2024 Physician-Scientist Trainee Diversity Summit conference proceedings

**DOI:** 10.1017/cts.2025.24

**Published:** 2025-02-11

**Authors:** Jessica Weng, Cynthia Y. Tang, Kyle T. Enriquez, Rohini N. Guin, Briana Christophers, Yentli E. Soto Albrecht, Daniel B. Amusin, Deborah D. Rupert, Paige Cooper Byas

**Affiliations:** 1 Mayo Clinic Medical Scientist Training Program, Mayo Clinic, Rochester, MN, USA; 2 University of North Carolina MD-PhD Program, University of North Carolina-Chapel Hill, Chapel Hill, NC, USA; 3 Vanderbilt University Medical Scientist Training Program, Vanderbilt University School of Medicine, Nashville, TN, USA; 4 Stony Brook University Medical Scientist Training Program, Stony Brook, NY, USA; 5 Cold Spring Harbor Laboratory, Cold Spring Harbor, NY, USA; 6 Weill Cornell/Rockefeller/Sloan Kettering Tri-Institutional MD-PhD Program, New York, NY, USA; 7 Perelman School of Medicine at University of Pennsylvania Medical Scientist Training Program, Philadelphia, PA, USA; 8 Medical Scientist Training Program, Northwestern University, Chicago, IL, USA; 9 Academic Scholars Advancement Program, Department of Anesthesiology, Washington University in St. Louis, St. Louis, MO, USA; 10 Burroughs Wellcome Fund, Research Triangle Park, NC, USA; 11 American Physician Scientists Association, Westford, MA, USA

**Keywords:** Physician-scientist development, diversity in medicine, human-centered design thinking, health equity

## Abstract

The Physician-Scientist Trainee Diversity Summit, hosted by the American Physician Scientists Association and the Burroughs Wellcome Fund, was conceived in 2019 with the mission of developing strategic plans to diversify the physician-scientist community using human-centered design thinking. In June 2024, the second iteration of this conference was held in Raleigh, North Carolina, and brought together a network of scientific and medical organizations to discuss issues of justice, equity, diversity, and inclusion facing physician-scientist trainees. This article summarizes the progress made from the first meeting, the proceedings of the 2024 Summit, and a thematic analysis of the recent meeting, offering tangible solutions to the physician-scientist community for supporting diversity and accessibility.

## Introduction

Physician-scientists serve important roles in healthcare innovation and the evolving landscape of medicine and science [1]. Their unique blend of clinical and research expertise enables them to bridge the gap between science and patient care, driving advancements that impact patient outcomes. Despite this, the physician-scientist workforce has been declining due to factors such as limited funding, prolonged training times, and barriers along the training pathway [2]. These barriers are often compounded for those who are underrepresented in science and medicine (UriSM), defined in this context as individuals who identify with a racial and ethnic background that is underrepresented in science and medicine relative to representation in the U.S. general population [3,4]. Similar barriers exist for trainees who are also primary caretakers, identify as having a chronic illness or disability, are first in their family to pursue higher education, identify as lesbian, gay, bisexual, transgender, and queer or questioning (LGBTQ+), come from lower socioeconomic backgrounds, or some combination thereof [5–9].

Addressing the needs of an increasingly diverse population in the United States (U.S.) requires fostering a diverse physician-scientist workforce [10]. Diversity in trainee and workforce backgrounds is critical for addressing health disparities that exist in the U.S. For example, patients have better outcomes when treated by physicians who share their racial or ethnic background [11,12]. Despite the benefits of a diverse workforce, those who identify as being from a UriSM background comprised 14% of all graduating MD-PhD students in 2023–2024 while over 30% of the U.S. population is from these backgrounds [13,14]. To discuss these issues and strategize solutions, the Burroughs Wellcome Fund (BWF) provided financial support to the American Physician Scientists Association (APSA) Physician-Scientist Trainee Diversity Working Group to host the first Physician-Scientist Trainee Diversity Summit in 2019. BWF is an independent private foundation whose mission is to “serve and strengthen society by nurturing a diverse group of leaders in biomedical sciences to improve human health through education and powering discovery in frontiers of greatest need.” BWF carries this out by funding research and career/professional development awards in defined focus areas as well as sponsoring complementary scientific and educational activities. APSA is the largest national organization representing physician-scientist trainees with over 2,000 members from all training stages from over 100 institutions across the U.S; its mission is to foster a community of physician-scientists in training, provide critical career-development resources, and represent the interests of physician-scientist trainees. The 2019 summit inspired several initiatives including the creation of the APSA Justice, Equity, Diversity, and Inclusion committee, the Virtual Summer Research Program (VSRP) for students looking for research opportunities during the COVID-19 pandemic, and increased research on the MD-PhD program pathway [15,16].

**Figure 1.**
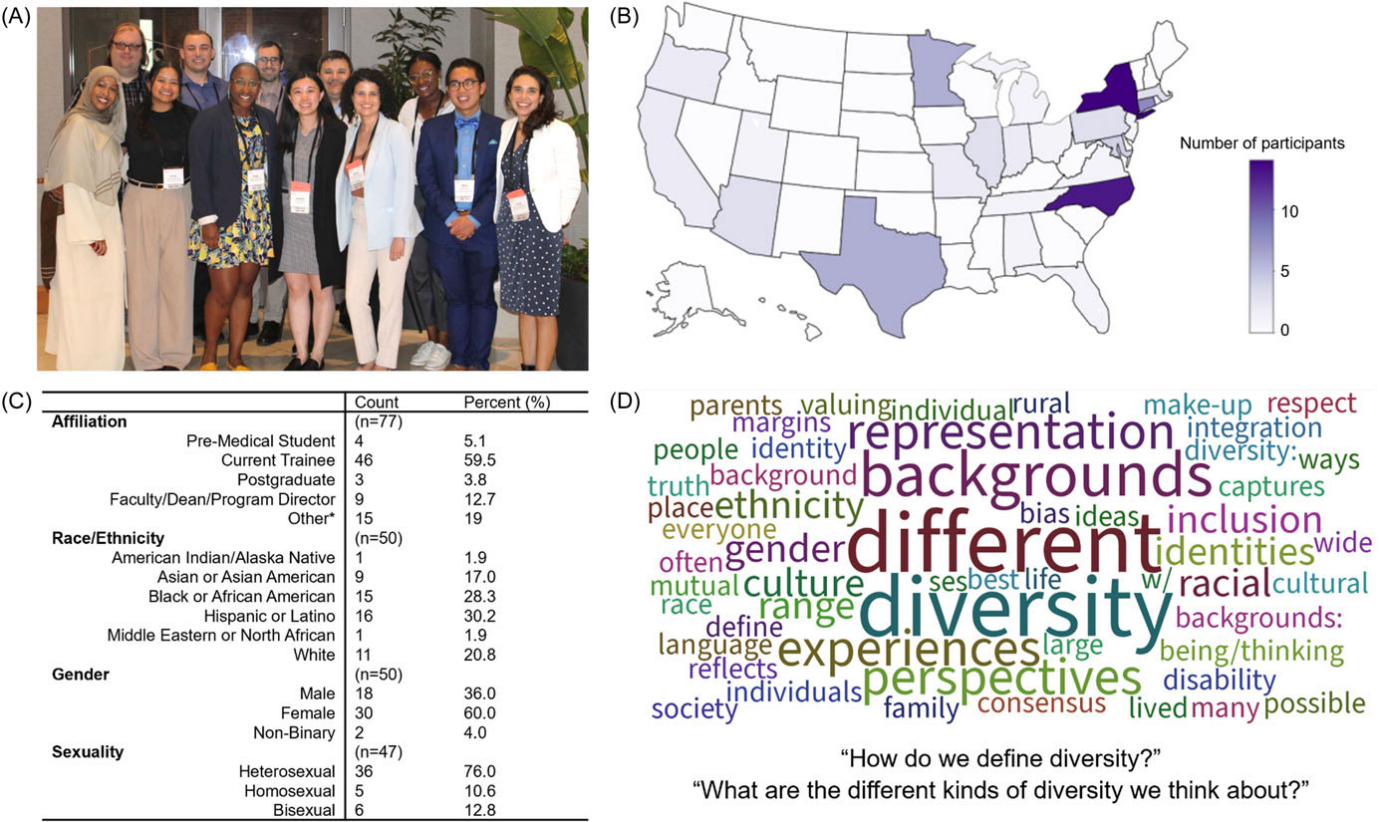
2024 Physician-Scientist Trainee Diversity Summit participants. (A) 2024 Diversity Summit volunteers, (B) geographic distribution of participants (*n* = 77), (C) participant demographics based on self-reported data, (D) participant responses to “How do we define diversity?” and “What are the different kinds of diversity we think about?” *Non-trainee organization representatives including Robert Wood Johnson Foundation, Building the Next Generation of Academic Physicians, National Institutes of Health, Longitudinal Evaluation of Research Career Intentions Among Medical Students (Underrepresented in medicine), American Association for the Advancement of Science, Research!America, Lasker Foundation, International Biomedical Research Alliance, American Association of Medical Colleges, Simons Foundation, and National Research Mentoring Network.

To build on the success of the first summit, APSA and BWF once again partnered to host the second Physician-Scientist Trainee Diversity Summit in 2024 (Figure 1A), which brought together representatives from affinity groups, faculty, MD-PhD program directors, and organizations invested in physician-scientist development (Figure 1B). Organizers aimed to gather individuals with established advocacy or scholarship in addressing barriers to physician-scientist training while ensuring that representation was diverse.

Both the 2019 and 2024 summits not only gave APSA an opportunity to convene its partners and discuss the important topic of diversity in the physician-scientist community but also enabled individuals from diverse backgrounds to support one another across training stages and learn about resources available to students at various institutions. Most participants in the 2024 summit reside in the Northeast or Southeast U.S., likely due to the Summit’s location in Raleigh, NC (Figure 1B). In the interest of maintaining access and opportunity for participation in this event, trainees and faculty across the pathway of physician-scientist development were recruited via APSA social media and newsletter-centered efforts. Nearly 60% of participants were in the undergraduate or medical education training stage (MD, DO, MD-PhD, DO-PhD students) (Figure 1C). All travel was supported by the BWF to ensure that funding was not a barrier. Participants were then prioritized based on career stage and geographical diversity. The mission of the Diversity Summit was to identify barriers and propose solutions to diversifying the physician-scientist workforce throughout training. Here, we report the approach, the resulting challenges identified, subsequent solutions, and current progress to further strengthen a more diverse physician-scientist workforce.

## Approach

Several affinity group organizations were invited to represent national perspectives on trainee experience and development. APSA views the inclusion of affinity groups as a significant advantage in discussions about diversity and the specific challenges encountered by individuals holding various identities. APSA is dedicated to integrating these perspectives into crucial conversations about the medical and graduate education for physician-scientist trainees in addition to understanding what diversity efforts already exist through these organizations which include, but are not limited to: the Student National Medical Association, Latino Medical Student Association, Asian Pacific American Medical Student Association, Association of Native American Medical Students, American Association of Black Physician Scientists, American Medical Women’s Association, Medical Student Pride Alliance, and American Medical Student Association.

The 2024 summit also gathered representatives from funding bodies and non-trainee-led organizations to participate in discussions about supporting physician-scientists-in-training from diverse backgrounds and sharing relevant opportunities. In attendance were representatives from the Robert Wood Johnson Foundation, Building the Next Generation of Academic Physicians, National Institutes of Health, Longitudinal Evaluation of Research Career Intentions Among Medical Students (Underrepresented in Medicine), American Association for the Advancement of Science, Research!America, Lasker Foundation, International Biomedical Research Alliance, American Association of Medical Colleges, Simons Foundation, and National Research Mentoring Network. Involvement of both governmental and private sectors in discussions of UriSM physician-scientists is critical to addressing the disparities in funding and career development, particularly as these organizations and the fellowships they support are critical drivers of physician-scientist careers.

The summit commenced by asking participants to reflect on “How do we define diversity? What are the different kinds of diversity we think about?” Participants’ responses provoked keywords including “representation,” “gender,” “ethnicity,” “culture,” “racial,” “disability,” “society,” and “margins” (Figure 1D). Through these identities, participants approached the Summit.

**Figure 2.**
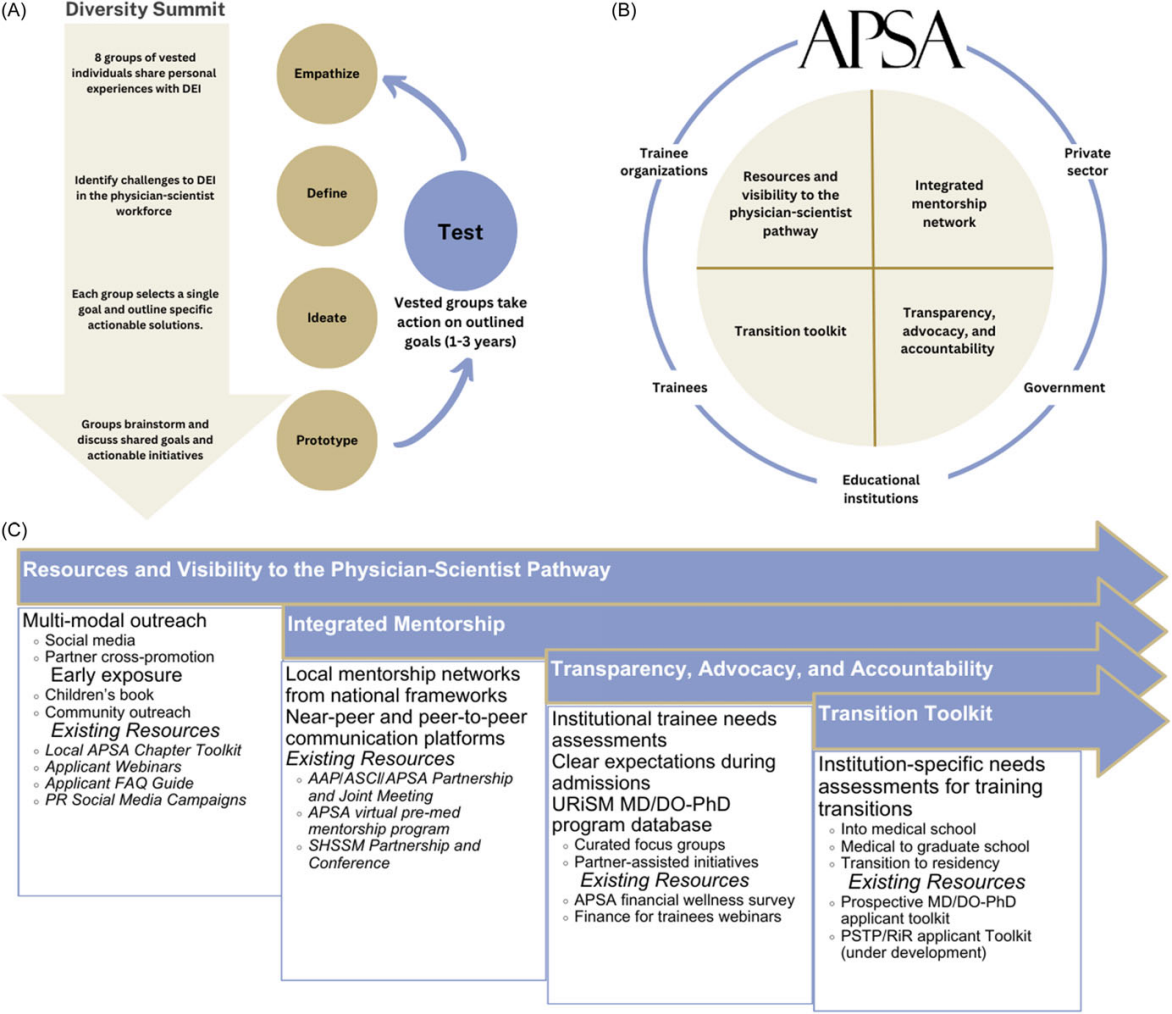
Human-centered design thinking (HCDT) at the Physician-Scientist Trainee Diversity Summit. (A) Description of the HCDT process throughout the diversity summit. (B) Groups identified four challenges and actionable solutions including 1) resources and visibility to the physician-scientist pathway, 2) an integrated mentorship network, 3) transparency, advocacy, and accountability, and 4) transition tool kit; these areas will be enacted upon by collaborations among American Physician Scientists Association, the private sector, government, trainees, educational institutions, and trainee affinity organizations.

Due to its success in 2019, human-centered design thinking (HCDT) approach was utilized again during the 2024 Summit (Figure 2A) [17]. HCDT is a structured process in which the “users,” in this case trainees and applicants, are the drivers of this process. This problem-solving process involves five phases: 1) empathize – participants share their personal DEI experiences and challenges, 2) define – participants identify shared challenges to DEI in the physician-scientist workforce, 3) ideate – participants select core ideas and outline actionable solutions, 4) prototype – participants brainstorm and discuss these shared goals and actionable initiatives, and finally, 5) test – vested organizations act on these ideas. Once these goals are tested, the plan is to reconvene at the next Diversity Summit. At the 2024 Summit, 77 participants were split into eight small groups of 8–10 participants to work through the 5 phases sharing out among the full group at the conclusion of each phase. The outcomes of the Diversity Summit are outlined below (Figure 2B).

## Challenges and proposed solutions (Figure 2B and 2C)

### Resources and visibility to the physician-scientist pathway

*Challenge*: Proactive recruitment is paramount for the physician-scientist pathway across all educational stages. Lack of early exposure to the physician-scientist career, starting as early as primary and secondary school and particularly for students at community colleges or institutions without affiliated medical schools presents a barrier to entering the pathway. Participants reported late awareness of dual-degree training programs and limited career counseling for UriSM students across training stages.

*Solution(s)*: Trainee-led organizations should address the lack of access to information by using multi-modal media outreach to promote the physician-scientist pathway with the goal of increasing interest throughout educational stages. Participants recognized that students rely on social media as a primary source of information and engagement. Therefore, social media outreach strategies could be used to show and depict diverse physician-scientists and trainees. Such strategies for engagement can be accomplished through social media challenges, cross-promoting content with partner organizations (including 2024 summit organizational representatives), and using local and regional chapters to increase local exposure to the physician-scientist path. Trainee-led organizations should also engage pre-health advisors to identify students who may be interested in the physician-scientist career path and provide resources for career guidance.

To target primary school-aged students, age-appropriate resources can be created, such as developing a children’s book featuring physician-scientist characters. Additionally, trainee organizations can develop a toolkit aimed at connecting with the local community by interacting with primary and secondary school-aged children to practice the scientific method and hypothesis testing to increase interest in science.

*APSA Activities to Address Challenge*: APSA has previously responded to this challenge by creating a toolkit for community engagement for local APSA chapters, free and interactive annual applicant webinars synchronous with the application cycle, and the VSRP during the COVID-19 pandemic. APSA has a list of pre-health advisor contacts at institutions, specifically focused on community colleges, historically Black colleges and universities, and institutions without an attached medical school to increase reach and awareness of APSA resources. APSA can also provide travel awards to pre-medical students to the annual Joint Meeting as well as regional meetings. Further, APSA has recently published the Applicant FAQ Guide which is freely available on the APSA website for dual-degree applicants. Finally, the APSA Public Relations Committee is now working on a Day in the Life series that showcases diverse physician-scientist trainees on our social media. APSA is committed to collaborating with our partners to expand our social media outreach and develop a children’s book about physician-scientists.

### Integrated mentorship network

*Challenge*: Students from UriSM backgrounds lack mentorship for scientific and clinical careers [18]. Participants identified that the physician-scientist training pathway is difficult to navigate, particularly for applicants interested in non-basic science disciplines, including the social sciences and humanities who may face additional barriers such as lack of funding, awareness, and opportunity [19]. Participants described a lack of access to and visibility of resources and mentorship in part due to a lack of a centralized platform for information-sharing and feedback on physician-scientist trainee-specific concerns.

*Solution(s)*: Trainees and curriculum development specialists can develop a framework for a mentorship program that can be adapted and implemented at individual institutions. Trainee-led organizations could also foster community by creating a nationwide online platform for information-sharing and connecting with peer and near-peer mentors.

*APSA Activities to Address Challenge*: APSA has partnered with the Association of American Physicians (AAP) and the American Society for Clinical Investigation (ASCI) to develop robust longitudinal and cross-sectional mentorship programs that culminate each year at the annual Joint Meeting, hosted by AAP, ASCI, and APSA. Further, APSA has a virtual mentorship program for premedical students that is supported by current dual-degree trainee volunteers, serving over 400 mentees annually. APSA furthers its commitment to this initiative by adding a near-peer mentorship session for the upcoming 2025 Joint Meeting as well as partnering with the Society for Humanities, Social Science, and Medicine during their biennial conferences.

### Transparency, advocacy, and accountability

*Challenge(s)*: MD/DO-PhD programs must increase transparency about their admissions and retention priorities, including information about financial support, health insurance, and training support, among other factors, to promote equity and enhance the success of prospective applicants. Lack of transparency leads to the need for strong individualized guidance from mentors or pre-health advisors to provide insight into program priorities, which is not accessible to all applicants. Though there are existing grassroots efforts to improve guidance and mentorship, applicants require increased transparency from dual-degree programs [5,15].

Of importance, equity for UriSM trainees necessitates financial literacy, accessibility, and stability. Socioeconomic status of pre-applicants, applicants, and trainees varies significantly [7,8]. Financial hardships vary throughout training and may affect access to academic resources in addition to personal expenses such as housing, food, childcare, healthcare, and emergency funds. Trainees need guidance about the financial options available to them over various phases of their careers to make informed decisions.

*Solution(s)*: Trainee-led organizations should conduct needs assessments with current and future trainees to understand what they prioritize in their program decisions, what information they wish they had before entering their training programs, and what they identified as factors contributing to their successes or setbacks. Similarly, institutions should report what application components are prioritized or most heavily weighted during their admissions processes. To address transparency and accountability, a database of MD/DO-PhD programs can be developed to include data on how they serve students from UriSM backgrounds, retention rates, and financial support.

*APSA Activities to Address Challenge*: APSA has developed a financial wellness survey for current trainees to understand financial barriers during each stage of training. These findings will be published publicly as a resource for trainees to make informed decisions while applying and for institutions to understand the financial challenges and priorities of trainees. APSA has also hosted interactive webinars on managing finances for dual-degree trainees. Further, APSA is creating focus groups to understand what trainees prioritize when selecting a dual-degree training program with the goal of using this information to collaborate with partners to create a database of dual-degree programs containing details about these priorities.

### Transition toolkit

*Challenge*: For retention of UriSM trainees, there is a need for professional development and transition support throughout training stages. Diversity Summit participants reported that specific trainee roadblocks such as disparate experiences, resources, and financial stability during transition points affect attrition rates, particularly for those who identify as UriSM [2,5–9].

*Solution*: Organizations could conduct a needs assessment to understand the specific trainee roadblocks at transition points in the physician-scientist pathway including choosing a program, transitioning between medical and graduate school, and transitioning to residency and fellowship. Subsequently, trainee-led organizations can assemble a transition toolkit available to all trainees and distributed through partners and mentorship networks.

*APSA Activities to Address Challenge*: APSA has developed a toolkit for prospective applicants that is publicly available on the APSA website. The Virtual Content Committee is leading the efforts this year to develop a similar transition guide for current trainees.

## Discussion

Diversity drives excellence and enhances innovation [20–22]. Intentionally increasing the breadth of lived experiences and knowledge base provides opportunities for learners to grow and challenge each other which is paramount in any learning environment. Furthermore, diversity in healthcare and science teams has been shown to improve patient outcomes, produce higher-quality science, and minimize abrasion in the face of change. Therefore, it is necessary to recruit and retain future generations of physician-scientists with a broad range of perspectives and experiences that will inform their worldviews and how they tackle biomedical problems. In turn, diversification of the biomedical workforce will help reduce healthcare disparities that exist throughout the U.S [20,23].

APSA, with support from BWF, organized the 2019 and 2024 Physician-Scientist Trainee Diversity Summits to create spaces to identify existing challenges and brainstorm novel solutions in the development of a diverse trainee population. The 2024 Diversity Summit groups identified gaps in the development and cultivation of a diverse physician-scientist workforce, which culminated into four distinct challenges and proposed solutions: resources and visibility to the physician-scientist pathway, integrated mentorship network, transparency, advocacy, and accountability of training programs, and a transition toolkit (Figure 2B, 2C). These discussions also revealed that much heterogeneity exists among programs and thus trainee experiences vary greatly in a program-specific manner, underscoring the need for institutions to work together in standardizing key elements that are fundamental to success during training.

However, despite the productive discussion we fostered, our ability to impact change has several limitations. First, as demonstrated by attendee data (Figure 1), equal representation across geographic regions was not obtained which may have skewed the perspectives provided at the conference; networks of communication for engaging trainees were primarily in states with active APSA chapters. Similarly, while attempts were made to prioritize trainee attendees, it remains the case that URiSM and women with caretaker duties are less likely to be able to attend such events, again skewing the voices presented. The ability to provide scholarship opportunities to cover travel, childcare, and conference fees is an important means of ensuring access to all academic conferences, including our own. Further, undergraduate trainee attendance was limited at the summit, yet as was pointed out during it, the need to address the diversity gap in the physician-scientist community begins by intervening at earlier stages of training. Therefore, access to the content of our conference (i.e., through website materials, recordings, or future hybrid options) may be a future goal of our organizations. Additionally, social media remains an important way to reach future generations and encourage teams with similar goals and audiences to capitalize on the diverse set of platforms available.

Nevertheless, herein we have showcased a strategy for other organizations to engage in diversity solution-generation through HCDT. After the 2024 Diversity Summit, APSA has developed a Diversity Summit Taskforce to continue the conversations and collaborate with partners to implement the identified solutions.

We call upon trainees, national organizations, and all others committed to diversifying the physician-scientist workforce to invest and participate in implementing the solutions outlined above. Engaging trainees in the identification of problems and the co-creation of solutions is an important step, and institutional leaders at academic institutions as well as in the private and public sectors that oversee the nation’s education system are critical in implementing practices and policies that can ensure the continued progress of diversifying the physician-scientist workforce. These efforts demand a concerted effort from all collaborators, and APSA leadership looks forward to progress on these goals.
